# *In Vivo* Tactile Stimulation-Evoked Responses in *Caenorhabditis elegans* Amphid Sheath Glia

**DOI:** 10.1371/journal.pone.0117114

**Published:** 2015-02-11

**Authors:** Gang Ding, Wenjuan Zou, Hu Zhang, Yadan Xue, Yang Cai, Guifang Huang, Lufeng Chen, Shumin Duan, Lijun Kang

**Affiliations:** 1 Department of Neurobiology, The Key Laboratory of Medical Neurobiology, The Ministry of Health of China, Zhejiang Provincial Key Laboratory of Neurobiology, Zhejiang University School of Medicine, Hangzhou, China; 2 Department of Pharmacology, Basic Medical College, Xinjiang Medical University, Urumqi, China; Australian National University, AUSTRALIA

## Abstract

Glial cells are important components of the nervous system. However, how they respond to physiological stimuli *in vivo* remains largely unknown. In this study, we investigated the electrophysiological activities and Ca^2+^ responses of the *C. elegans* amphid sheath glia (AMsh glia) to tactile stimulation *in vivo*. We recorded robust inward currents and Ca^2+^ elevation in the AMsh cell with the delivery of tactile stimuli of varying displacements to the nose tip of the worm. Compared to the adjacent mechanoreceptor ASH neuron, the AMsh cell showed greater sensitivity to tactile stimulation. Amiloride, an epithelial Na^+^ channel blocker, blocked the touch-induced currents and Ca^2+^ signaling in the ASH neuron, but not those in the AMsh cell. Taken together, our results revealed that AMsh glial cells actively respond to *in vivo* tactile stimulation and likely function cell-autonomously as mechanoreceptors.

## Introduction

Glial cells are important components of the nervous system. They contribute to the development, function and health of neurons [1].

It is now widely believed that glial cells are not only supportive, but also take part actively in the modulation of neuronal activity. For instance, astrocytes, the most numerous glial cells in the vertebrate central nervous system, can form the “tripartite synapse” with adjacent pre- and postsynaptic terminals [1–3]. Neurotransmitters released from presynaptic terminals activate not only postsynaptic terminals, but also astrocytes. Subsequently, astrocytes release gliotransmitters such as glutamate, ATP, and D-serine which may participate in synaptic events and long-term plasticity [1–3]. However, previous studies were mainly based on cultured cells and brain slices. It remains largely elusive how glial cells respond to physiological stimuli *in vivo* to regulate neuronal activity in their natural environment [2–4].

*Caenorhabditis elegans* has a simple nervous system, consisting of 302 neurons and 56 glial cells. Nonetheless, it performs a diversity of behaviors, from simple, such as avoidance, to complex, such as social feeding, mating, drug addiction and a certain degree of learning and memory [5,6]. Previous studies have suggested *C*. *elegans* glial cells are developmentally, morphologically, and functionally analogous to those of vertebrates [5,7]. For example, UNC-6/Netrin secreted from the cephalic sheath glia (CEPsh glia) are required for proper guidance of the synapse formation between AIY and RIA neurons in *C*. *elegans* [8,9]. Glial DEX-1 is required for amphid sensory dendrite extension [10]. Amphid sheath glial cells (AMsh glial cells) guide the morphological remodeling of AWC neurons in dauer worms [11].

The amphids, the largest sensory organs in *C*. *elegans*, are bilaterally located in the head. Each amphid organ consists of twelve sensory neurons and two glial cells, a sheath cell (AMsh glial cell) and a socket cell [5]. These specialized sensory neurons detect various environmental stimuli and lead to attractive and repulsive behaviors. The amphid sensory neuron ASH is the main nociceptor in *C*. *elegans*. It is a polymodal neuron mediating avoidance responses to a wide range of noxious stimuli, such as tactile stimulation (nose touch), volatile repellent chemicals (1-octanol), heavy metals (Cu^2+^) and hyperosmolarity (glycerol) [12]. Notably, ASH is the only mechano-receptor neuron in the amphid. AMsh glial cells are physically associated with the amphid sensory neurons [5,13]. Laser ablation of AMsh glial cells in embryonic stage caused defects in dendrite formation of amphid sensory neurons [5,7]. Taulant et al. (2008) elegantly revealed that AMsh glial cells are also essential for multiple aspects of sensory organ function [7]. They found that the Ca^2+^ elevation in the ASH neuron in response to environmental stimuli is abolished after the AMsh glial cell is laser-ablated at the larval stage, even though no detectable structural change occurs[5,7]. Other studies have also indicated that glial cells are involved in the modulation of neuronal function in *C*. *elegans*. A recent study by Lu *et al*. identified two DEG/ENaC channel subunits, DELM-1 and DELM-2, in the socket glial cells associated with the touch-receptor neurons OLQ and IL[14]. Their data suggest that DELM-1 and DELM-2 function in these glial cells to modify touch-avoidance behavior in *C*. *elegans*[14].

These studies support the essential roles of glial cells in the *C*. *elegans* nervous system. However, the underlying molecular and circuit mechanisms remain poorly understood. Therefore, in this study, we applied *in vivo* patch clamp recording and Ca^2+^ imaging to monitor tactile stimulation-evoked activities in the AMsh glial cell, and compared these responses to those in the ASH neuron.

## Materials and Methods

### *C*. *elegans* strains

The strain used was wild-type strain N2. Worms were well-fed on NGM plates seeded with *Escherichia coli* at 20°C using standard methods [15]. Day 2 adult worms were used in all experiments.

### Transgenes

All expression plasmids are based on the pPD95.75 backbone. Standard methods were used to generate all plasmids. We generated transgenic worms by microinjection with plasmids. P*vap-1*::*mCherry* and P*sra-6*::*DsRed* were injected at a concentration of 20 ng/μl, while P*vap-1*::*GCaMP5*.*0*, P*sra-6*::*GCaMP5*.*0*, and P*str-3*::*YFP* were injected at 80 ng/μl.

### *In vivo* electrophysiology

Whole-cell patch clamp recordings were performed on an Olympus microscope (BX51WI) with an EPC-10 amplifier and the Patchmaster software (HEKA) as previously described [16, 17]. Signals were filtered at 2 kHz, sampled at 20 kHz, and analyzed using IGOR Pro software (Wavemetrics). Recording pipettes were pulled from borosilicate glass capillaries (B-120–69–10, Sutter Instruments) to a resistance of 10–15 MΩ on a P-97 micropipette puller (Sutter Instruments). Day 2 worms were glued on the surface of a sylgard-coated cover glass using a medical-grade, cyanoacrylate-based glue (Gluture Topical Tissue Adhesive, Abbott Laboratories) and dissected as previously described [16, 18]. Briefly, a piece of cuticle in the head of the worm, approximately 100 x 40 μm, was cut open using a sharp glass pipette with a tip O.D. of ∼0.2 μm. The cut-edge of the cuticle was glued down to the coverslip to expose the cell body of the AMsh glial cell and the ASH neuron, which were identified by the ﬂuorescent markers expressed by P*vap-1*::*mCherry* and P*sra-6*::*DsRed* (ASH, ASI)+P*str-3*::*YFP*(ASI), respectively. Touch stimuli were delivered to the nose tip of the worm using a tip diameter of ∼5 μm borosilicate glass capillary driven by a piezoelectric actuator (PI) mounted on a micromanipulator (Sutter). The bath solution contained (in mM) 145 NaCl, 2.5 KCl, 1 CaCl_2_, 1 MgCl_2_, 20 D-glucose and 10 HEPES (325∼335 mOsm, pH adjusted to 7.3). The pipette solution contained (in mM)145 Cs-gluconate, 2.5 KCl, 5 MgCl_2_, 10 HEPES, 0.25 CaCl_2_, 10 glucose, 5 EGTA, 5 Na_2_ATP and 0.5 NaGTP (315∼325 mOsm, pH adjusted to 7.2). For high Cl^-^ experiments, Cs-gluconate was replaced with CsCl in pipette solution. Membrane potentials were clamped at-70 mV.

### Calcium Imaging

Ca^2+^ imaging was performed on an Olympus microscope (BX51WI) with a 60×objective lens, an Andor DL-604M EMCCD camera, and μManager software (developed in Ron Vale's laboratory at UCSF based on ImageJ). The genetically-encoded Ca^2+^ sensor GCaMP5.0 was expressed in the AMsh glial cell and the ASH neuron by the *vap-1* and the *sra-6* promoter, respectively[21]. The Ca^2+^-insensitive fluorescent protein mCherry was co-expressed with GCaMP5.0 as a reference. ΔR/R was used to quantify the fluorescence changes (R = F_GCaMP5.0_/ F_mCherry_).

### Statistical analysis

Data analysis was performed using Excel 2010 (Microsoft) and Igor Pro 5.0 (Wavemetrics). Error bars were mean ± SEM. n represents the number of cells. P values were determined by Student’s *t* test. P < 0.05 was regarded as statistically significant.

## Results

### *In vivo* tactile stimulation evokes robust inward currents in the AMsh glial cell

**Fig 1:**
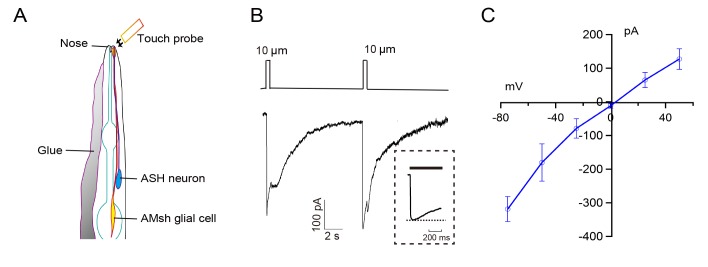
*In vivo* patch-clamp recording of touch-induced currents from the *C*. *elegans* AMsh glial cells. (A) Diagram of the morphologies of the AMsh cell and the ASH neuron [5, 13]. Tactile stimuli were delivered to the nose tip of the worm using a ∼ 5μm glass probe. (B) Representative currents in an AMsh cell evoked by a pair of tactile stimuli (500 ms). Inset shows the period of tactile stimulation in expanded time scale. (C) I-V curve of the touch-induced currents in the AMsh cells. Displacement,10 μm; mean ± s.e.m, n≥5 animals; Voltage clamped at-70 mV.

To measure touch-evoked changes, we applied *in vivo* whole-cell patch clamp recording to the AMsh glial cell, which was identified with transgenic fluorescent protein mCherry under the AMsh cell-specific promoter *vap-1*[7]. In our transgenic strain, both cell body and process of the AMsh cell were well-labeled. The AMsh cell process is located in the nose tip of the worm and surrounds the proximal regions of the sensory cilia of amphid sensory neurons such as the ASH neuron [5, 7] (**Fig. 1A**). To expose the AMsh cell body for whole-cell recording, a day 2 adult (D2) worm was glued on sylgard-coated coverslip under bath solution, then a small piece of cuticle on the head of the worm was carefully cut opened and glued down to the coverslip to expose the cell body for recording [16,18]. The nose tips of all dissected worms used in recording remained intact. Any accidentally damaged preparations were discarded.

Tactile stimuli applied to the nose tip of the worm evoked robust inward currents in the AMsh cell **(Fig. 1B)**. Similar to previous observations of mechano-receptor currents in *C*.*elegans* touch-receptor neurons CEP, PDE, PVD, PLM and ASH [16,18,22,23], the evoked currents in the AMsh cell adapted while stimulation was maintained. The amplitude of the current after 500 ms of sustained tactile stimulation was 74.0± 4.7% of the peak value (n = 6). Occasionally, a smaller "off" current was observed with the removal of the stimulation.

We then examined I-V relationships of touch-evoked currents in the AMsh cell. Similar to mechano-receptor currents in all previously recorded touch-receptor neurons, touch-evoked currents in the AMsh cell were inward when the cells were negatively voltage clamped [16, 18, 22, 23]. As we previously reported mechano-receptor currents in CEP and PDE neurons, which are mediated by a mechano-sensitive N-type TRP (NOMPC) subfamily channel protein TRP-4, are non-selective to small cations such as Na^+^, Ca^2+^, K^+^, and Cs^+^, and show a reversal potential at 0 mV [16,18]. On the other hand, the mechano-receptor currents in PLM, PVD and ASH neurons are mediated by touch-sensitive degenerin/epithelial Na^+^ family channels (DEG/ENaC), such as MEC-4 and DEG-1.They were highly Na^+^-selective and amiloride-sensitive with reversal potentials at +50∼+70 mV [18,22,23]. In this study, we found that the reversal potential of touch-evoked currents in the AMsh cell was ∼0 mV (**Fig. 1C**). The reversal potential of touch-evoked currents in the AMsh cell was not affected by replacement of gluconate in the pipette solution with Cl^-^ **(S1 Fig.)**.

### The amplitudes of touch-evoked currents in the AMsh glial cell are stimulus strength-dependent

**Fig 2:**
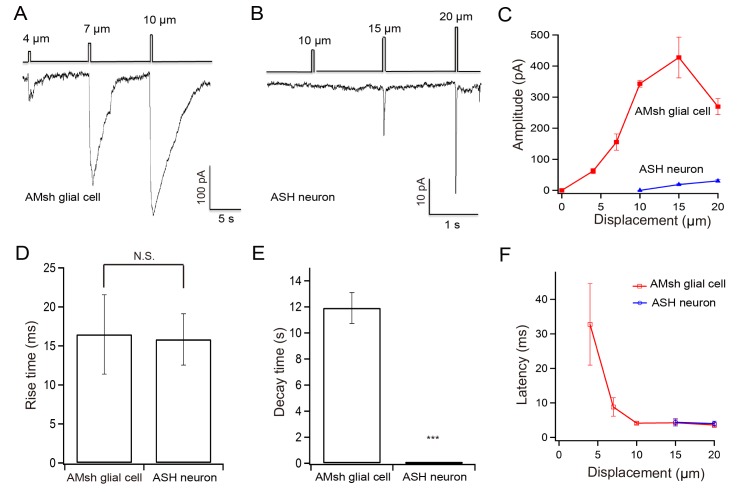
Sensitivities of the AMsh cell and the ASH neuron to stimulus strength. Tactile stimuli of varying displacements were applied to the nose tip of the worm. (A) Touch-induced currents in an AMsh cell evoked by 4, 7, and 10 μm displacements. (B) Inward currents in an ASH neuron evoked by 10, 15, and 20 μm displacement. (C) Amplitudes of touch-induced currents in the AMsh cells and the ASH neurons. A displacement of 4μm was able to evoke currents in the AMsh cells, whereas a displacement <10μm failed to evoke currents in the ASH neurons. (D) Rise-times of currents evoked by a 15 μm displacement. (E) Decay-times of touch-induced currents evoked by a 15 μm displacement. The decay time is measured as the time elapsed for the current to fully recover from the peak. (F) Latencies of touch-induced currents in the AMsh cells evoked by 4, 7, 10, and 15 μm displacement and ASH neurons evoked by 15 and 20 μm displacement. Mean ± s.e.m, n≥4 animals; Voltage clamped at-70 mV.

Tactile stimuli of varying displacements were applied to the nose tip of the worm. Occasionally, a displacement of 4 μm induced small currents in the AMsh cell. We recorded robust touch-evoked currents with a displacement of 10 μm. The amplitude of touch-evoked currents increased in proportion to displacement in the range of 4 to 15 μm, then it saturated (4 μm *vs* 7μm, P = 0.028*; 4 μm *vs* 10μm, P = 0.0003***; 7 μm *vs* 10μm, P = 0.027*) (**Fig. 2A,C,** and **S2 Fig**). Similar results have been previously reported in CEP, PDE, PVD, PLM and ASH neurons, though the stimulus strengths required to evoke these neurons differ [16,18,22,23].

### The AMsh glial cell is more sensitive to tactile stimulation than its adjacent touch-receptor ASH neuron

One possibility is that the currents we recorded in the AMsh cell were not directly evoked by tactile stimulation, but indirectly induced by activation of its adjacent touch-receptor neurons. Sensory cilia of several mechanoreceptor neurons including CEP, ASH, OLQ, IL1, and FLP are located in the nose tip of the worm. The dopaminergic neuron CEP mainly detects fine bacterial particles, and contributes to the basal slowing response [13,17]. The ASH, OLQ, and FLP neurons are responsible for nose touch-avoidance behaviors [13]. OLQ and IL1 are thought to mediate the aversive head-withdrawal reflex [14]. Touch-evoked currents in the AMsh cell are unlikely to be due to the OLQ, FLP, IL1 and CEP neurons, because these neurons are neither located in the amphid nor physically connected to the AMsh cell [5]. The ASH neuron is the only mechano-receptor neuron in the amphid and is closely associated with the AMsh cell [5,7,13]. For comparison, we recorded touch-evoked responses in the ASH neuron. Consistent with its function as a nociceptor, we found the threshold required for triggering touch-evoked currents in the ASH neuron was ∼10 μm (Fig. 2B-C). Shana *et al*. also reported more forces were required to activate touch-evoked currents in the ASH neuron than in the PLM neuron, which is a gentle touch receptor (**S1 Table**)[20]. These findings indicate that the AMsh cell is more sensitive to tactile stimulation than the ASH neuron.

The amplitude of touch-evoked currents in the AMsh cell (427.67± 65.74 pA at 15 μm displacement; n = 6) was much larger than that in ASH neuron (30.29± 4.03 pA at 20 μm displacement; n = 6)(**Fig. 2C**). The amplitudes of all previously reported touch-receptor currents in *C*. *elegans* sensory neurons were close to the value we recorded here in the ASH neuron (**S1 Table**) [16,17,20,21]. Thus, touch-evoked currents in the AMsh glial cell are robust.

### Touch-induced currents in the AMsh glial cell show fast activation and slow decay kinetics

Fast activation kinetics is a common feature of mechano-gated channels [17,20]. In our recording system, a displacement of 15 μm took 16.47±5.09 milliseconds (n = 6) to fully activate touch-induced currents in the AMsh cell and 15.83± 3.28 milliseconds (n = 6) in the ASH neuron (**Fig. 2D**). The rise-times of the touch-induced currents in these two types of cells were not significantly different. However, the decay rates were markedly distinct. It took 11.91±1.18 seconds in the AMsh cell and 55.84± 7.46 milliseconds in the ASH neuron for touch-induced currents to fully recover from the peak (15μm displacement; n = 6) **(Fig. 2E)**. The molecular basis and physiological meanings of slow decay of touch-induced currents in the AMsh cell are unknown. Some channel(s) with slow decay kinetics may be activated by tactile stimulation in the AMsh cell and lead to long-lasting ion flux. As a consequence, the AMsh cell may modulate neuronal activity through long-lasting release of gliotransmitters.

### Touch-induced currents in the AMsh glial cell show short latency

Mechano-gated channels can be activated directly by forces. This process is not dependent on intracellular second messengers [22]. Thus the latency, which is the time between stimulus delivery and channel activation, is shorter than that for second-messenger pathways [17,20,22]. So far the latency of fastest known second messenger-mediated sensory transduction is 5∼20 ms [17,20]. Our data show that the latency of touch-induced currents in the AMsh cell decreased with increasing stimulus strength. With a displacement of 15μm, the latency was 4.3± 0.9 ms (n = 6) in the AMsh cell and 4.1± 1.1 ms (n = 6) in the ASH neuron (**Fig. 2F**). Thus, it is unlikely that second messengers are required for activation of the AMsh cell.

### *In vivo* tactile stimulation evokes a robust Ca^2+^ increase in the AMsh glial cell

**Fig 3:**
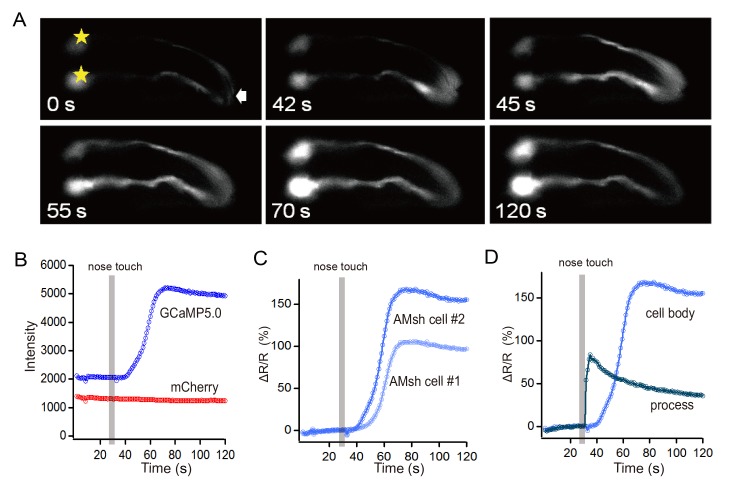
*In vivo* tactile stimulation induced Ca^2+^ elevation in *C*. *elegans* AMsh cell. (A) An example of intracellular Ca^2+^ elevation in an AMsh cell induced by a train of tactile stimuli (20 μm, 2 Hz, 5 s). Sample times are indicated in seconds. *, cell body of the AMsh cell. (B) Fluorescence intensities in the cell body of an AMsh cell. (C) Ratio changes in the cell bodies of the AMsh cells. (D) Ratio changes in the process and cell body of an AMsh cell. The Ca^2+^ wave was propagated from the process to the cell body.

Ca^2+^ signaling is the hallmark of astrocyte activation [1,23]. To test whether touch-induced currents can substantially activate the AMsh cell, we monitored its intracellular Ca^2+^ level using a Ca^2+^-sensitive fluorescent protein GCaMP5.0 [19]. GCaMP5.0 and mCherry were co-expressed in the AMsh cell using the *vap-1* promoter. Consistent with our electrophysiological data, we found a robust Ca^2+^ elevation in the AMsh cell by touching the nose tip of the worm. Remarkably, the Ca^2+^ signal clearly propagated from the distal ending of the process in the nose tip to the AMsh cell body (**Fig. 3A-D; S1 Movie**). The delay of Ca^2+^ elevation in the cell body of the AMsh cell was 13 ± 1 seconds (n = 10). The protocol we used to touch the nose tip of the worm was a train of stimuli with 20 μm displacement (2 Hz, 5 s). Tactile stimulation with 10 μm displacement induced a weaker Ca^2+^ elevation in the AMsh cell (ratio increased 12.12± 7.02% with 10 μm and 54.98± 8.87% with 20 μm; n≥4; **S3 Fig.; S2 Movie**).

### Touch-induced responses in the AMsh glial cell are independent of the ASH neuron and amiloride-sensitive ENaC channels

**Fig 4:**
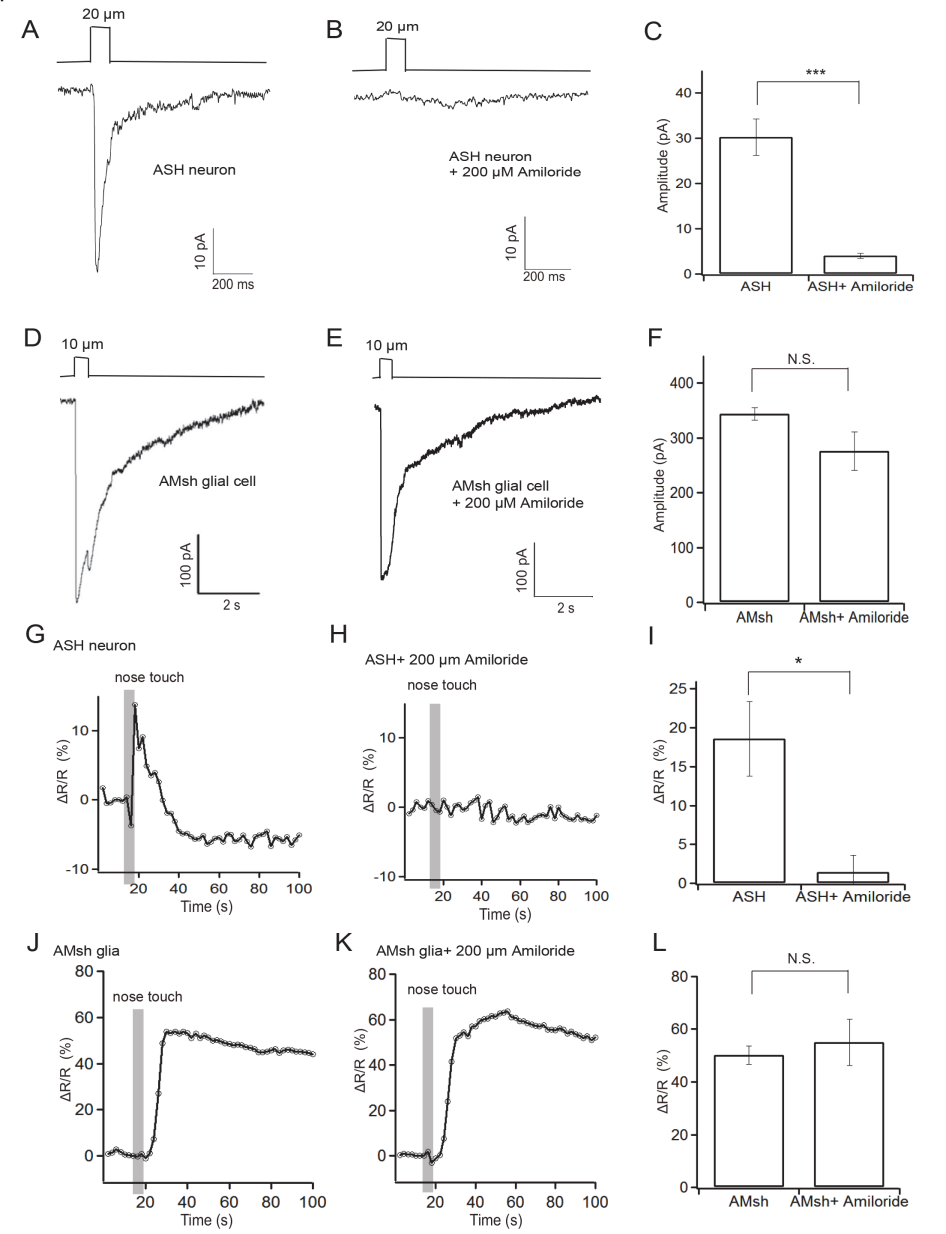
Effect of amiloride on touch-evoked responses in the AMsh glial cell and the ASH neuron. (A) Representative recording from an ASH neuron (20 μm displacement; voltage clamped at-70 mV). (B) Representative recording from an ASH neuron with 200 μM amiloride in the bath solution. (C) Statistical summary of data as in (A) and (B). (D) Representative recording from an AMsh cell (10μm displacement; voltage clamped at-70 mV). (E) Representative recording from an AMsh cell with 200 μM amiloride in the bath solution. (F) Statistical summary of data as in (D) and (E).(G) Representative GCaMP/mCherry ratio increase in an ASH neuron induced by tactile stimulation (20 μm, 2 Hz, 5 s). (H) Representative GCaMP/mCherry ratio increase from an ASH neuron with 200 μM amiloride in the bath solution. (I) Statistical summary of data as in (G) and (H). (J) Representative GCaMP/mCherry ratio increase in an AMsh cell induced by tactile stimulation (20 μm, 2 Hz, 5 s). (K) Representative GCaMP/mCherry ratio in an AMsh cell with 200 μM amiloride in the bath solution. (L) Statistical summary of data as in (G) and (H). Mean ± s.e.m, n≥4 animals. ΔR/R was used to quantify the fluorescence changes (R = F_GCaMP5.0_/ F_mCherry_).

Astrocytes can be activated by pre-synaptic terminals [23]. We therefore asked if the activity of the AMsh cell evoked by tactile stimulation is mediated by the ASH neuron. Touch-induced currents from the ASH neuron are mainly carried by the DEG/ENaC channel protein DEG-1, which can be blocked by amiloride [20]. We found both the touch-evoked currents and Ca^2+^ signaling in the ASH neuron were abolished by application of 200μM amiloride in the bath solution **(Fig. 4D-F, J-L; S3, S4 Movies).** However, amiloride did not affect the currents and Ca^2+^ elevation in the AMsh cell (**Fig. 4A-C, G-I; S5 Movie)**. Thus amiloride-sensitive ENaC channels such as MEC-4 and DEG-1 are not essential for the touch-evoked responses in the AMsh cell. Meanwhile, our observations suggest that the touch-evoked responses in the AMsh cell are independent of the ASH neuron.

## Discussion

In this study, we found *in vivo* tactile stimulation evoked robust inward currents and a Ca^2+^ increase in the *C*. *elegans* AMsh glial cell. These currents had a reversal potential at 0 mV. Considering that the AMsh glial cell is physically associated with the mechanoreceptor ASH neuron in the amphid organ [7,13], we compared their responses to tactile stimulation. Our data show that the threshold of stimulus strength required to evoke a response in the AMsh cell was lower than that in the ASH neuron. Both the touch-induced currents and Ca^2+^ increase in the ASH neuron were blocked by amiloride, while those in the AMsh cell were amiloride-insensitive, suggesting that the AMsh cell may cell-autonomously serve as a mechanoreceptor.

Touch-induced currents in the AMsh cell were unlikely induced by mechanoreceptor neurons. First, the only touch-receptor neuron closely associated with the AMsh cell in *C*. *elegans* is the ASH neuron [7,13]. Our data show that the AMsh cell is more sensitive to tactile stimulation than the ASH neuron. Thus activation of the AMsh cell does not rely on activation of the ASH neuron. Second, the latency of touch-induced currents in the AMsh cell was very short, 4.3±0.9 ms with a displacement of 15 μm, which is as fast as activation of the ASH neuron and is faster than any known second-messenger pathway. It indicates that the AMsh cell and the ASH neuron are activated simultaneously by tactile stimulation. Third, the blockade of touch-evoked currents and Ca^2+^ signaling in the ASH neuron by amiloride did not affect the touch-induced response in the AMsh cell. In conclusion, an appealing model is that the AMsh glial cell serves cell-autonomously as a touch receptor.

Two classes of mechano-gated channels have been identified in *C*. *elegans*. One is amiloride-sensitive ENaC channels such as DEG-1 and MEC-4. Another is the N-type TRP channel TRP-4, which is expressed in dopaminergic neurons and is resistant to amiloride [16,17,20–22,24]. Our data show that ENaCs are not required for touch-evoked activities in the AMsh cell, because amiloride did not block the responses. Since TRP-4 is not expressed in the AMsh cell, and TRP-4-expressing CEP/ADE/PDE neurons are not physically connected with the AMsh cell [5,25], TRP-4 may not be the channel mediating the touch-evoked responses. To date, only three classes of ion channels including DEG/ENaC, TRPN and Piezo have been proven to be mechano-gated channels in eukaryotes [17, 25, 26, 27]. A variety of other channels including the TRP family proteins (such as TRPA1, TRPV4, PKD2, NANCHUNG and INACTIVE), the transmembrane channel-like family proteins (TMC1, TMC2), and the stretch-sensitive two pore-domain K^+^ channels (K_2_P channels such as TREK-1and TRAAK) have also been implicated in mechanosensation [17, 25, 26, 27]. It would be interesting to reveal the identity of the channel(s) mediating the touch-induced responses in the AMsh glial cell. Piezos, TMCs, TRPs and K_2_Ps are among the most promising candidates. Meanwhile, we do not exclude novel channel(s) at this stage.

It is noteworthy that powerful *in vivo* functional assays, such as electrophysiology, Ca^2+^ imaging, and optogenetics, have been used in the study of neurons, but have barely been employed in the study of glial cells in *C*. *elegans* [17, 28, 29]. In a previous study, GCaMP2.0 was employed to investigate the Ca^2+^ signaling in the cultured *C*. *elegans* cephalic sheath (CEPsh) glial cell, whereas *in vivo* Ca^2+^ signaling in this cell has yet to be addressed[30]. Given that *in vivo* studies of astrocytes and neuron-glia interactions in mice are challenging[2–4], *C*. *elegans* may offer a valuable model to dissect the mysterious functions of glia and to reveal the core mechanisms of neuron-glia interactions in their natural environment, taking advantage of the rapid genetics and powerful *in vivo* functional assays in this classic model organism.

## Supporting Information

S1 Table Comparison of the touch-induced currents in the AMsh glia cell with those in mechano-receptor neurons. (TIF)

S1 Fig I-V curve of touch-induced currents in AMsh glial cells recorded with high Cl^-^ solution in the pipette. Cs-gluconate was replaced with CsCl in the pipette solution (10 μm displacement; mean ± s.e.m, n = 5 animals; voltage clamped at-70 mV). (TIF)

S2 Fig Representative touch-induced currents in an AMsh glial cell evoked by 15 and 20 μm displacement, respectively (voltage-clamped at-70 mV). (TIF)

S3 Fig Tactile stimulation-induced Ca^2+^ elevation in an AMsh glial cell. (A)Representative GCaMP/mCherry ratio changes induced by 10 μm displacement (2 Hz, 5 s). (B)Statistical summary of GCaMP/mCherry ratio changes induced by 10 μm and 15 μm displacement (2 Hz, 5 s) (mean± s.e.m.; n≥4).
(TIF)

S1 Movie *In vivo* tactile stimulation-induced Ca^2+^ elevation in an AMsh glial cell. The Ca^2+^ level was monitored by GCaMP5.0 expressed in the AMsh cell. 20 μm displacement, 2 Hz, 5 s.
(WMV)

S2 Movie *In vivo* tactile stimulation-induced Ca^2+^ elevation in an AMsh glial cell. The Ca^2+^ level was monitored by GCaMP5.0 expressed in the AMsh cell. 10 μm displacement, 2 Hz, 5 s.
(WMV)

S3 Movie *In vivo* tactile stimulation-induced Ca^2+^ elevation in an ASH neuron. The Ca^2+^ level was monitored by GCaMP5.0 expressed in the ASH neuron. 20 μm displacement, 2 Hz, 5 s.
(WMV)

S4 Movie *In vivo* tactile stimulation-induced Ca^2+^ elevation in ASH neuron is blocked by 200 μM amiloride. The Ca^2+^ level was monitored by GCaMP5.0 expressed in the ASH neuron. 20 μm displacement, 2 Hz, 5 s.
(WMV)

S5 Movie *In vivo* tactile stimulation-induced Ca^2+^ elevation in the AMsh cell is resistant to 200 μM amiloride. The Ca^2+^ level was monitored by GCaMP5.0 expressed in the AMsh cell. 20 μm displacement, 2 Hz, 5 s.
(WMV)
