# Supplementary figures and images for: In Vivo Tactile Stimulation-Evoked Responses in Caenorhabditis elegans Amphid Sheath Glia
Source: PLoS One. 2015 Feb 11;10(2):e0117114. doi: 10.1371/journal.pone.0117114 (PMC4325002; doi:10.1371/journal.pone.0117114)

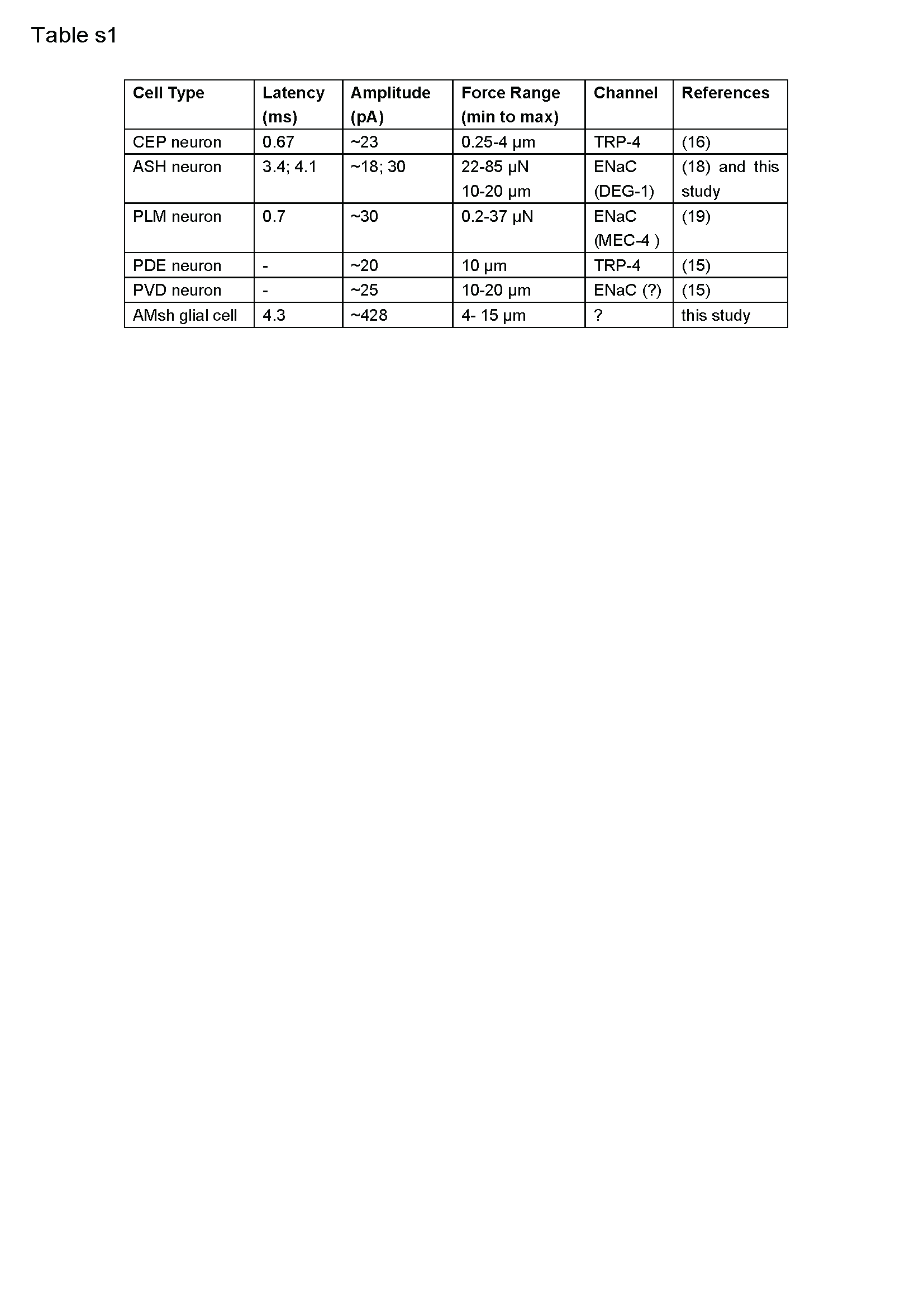

Supplement: S1 Table — (TIF) [file pone.0117114.s001.tif]

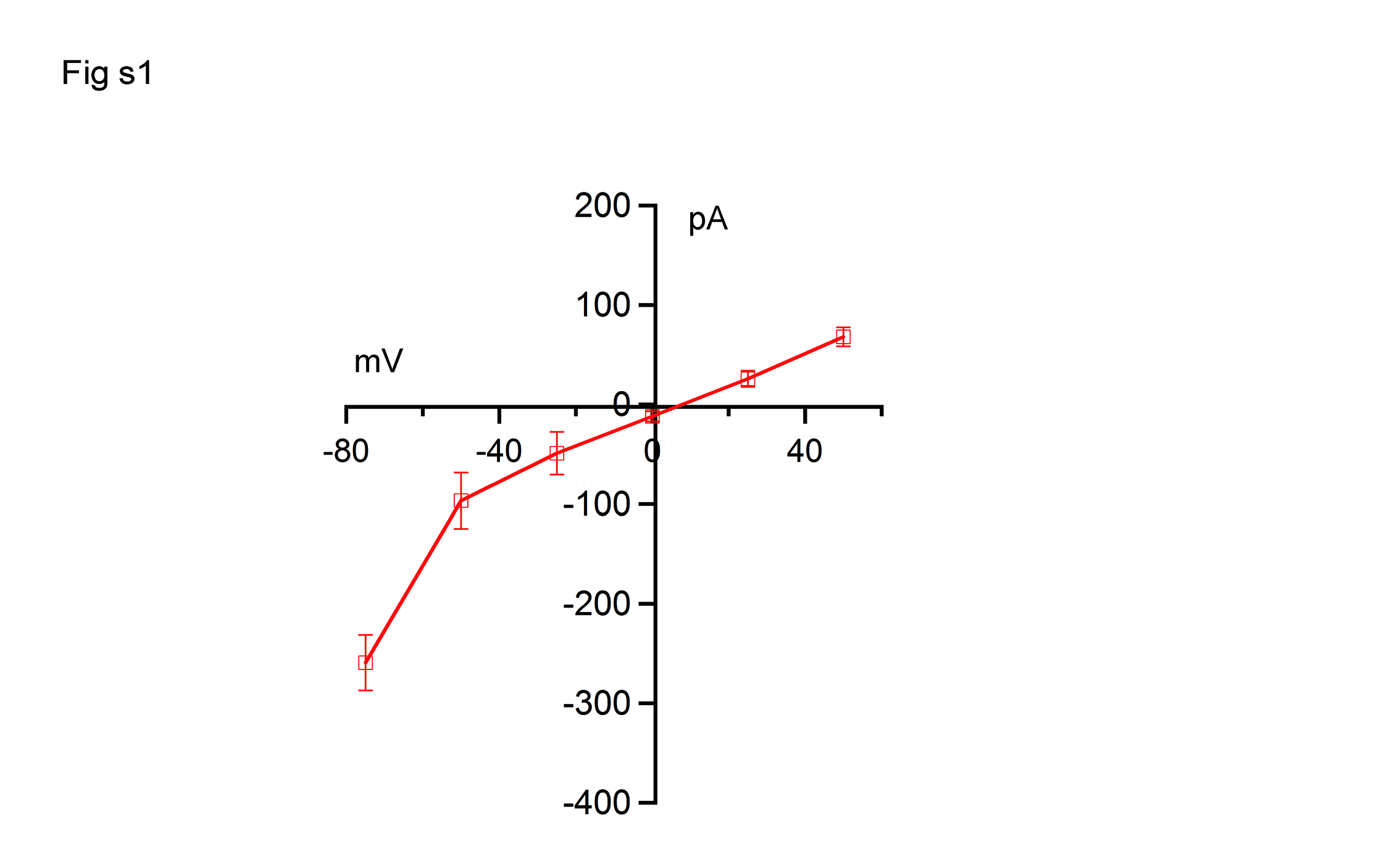

Supplement: S1 Fig — (TIF) [file pone.0117114.s002.tif]

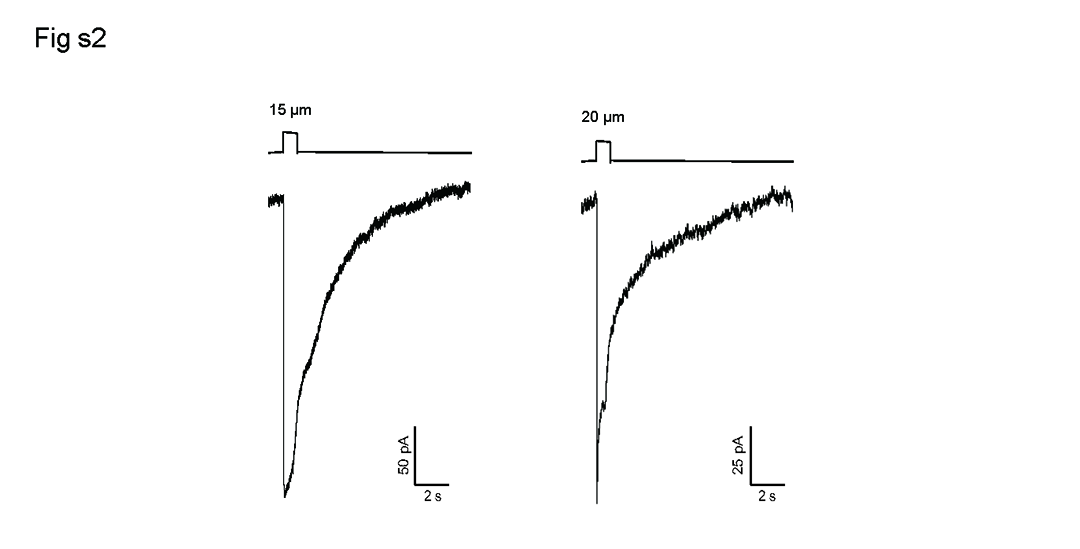

Supplement: S2 Fig — (TIF) [file pone.0117114.s003.tif]

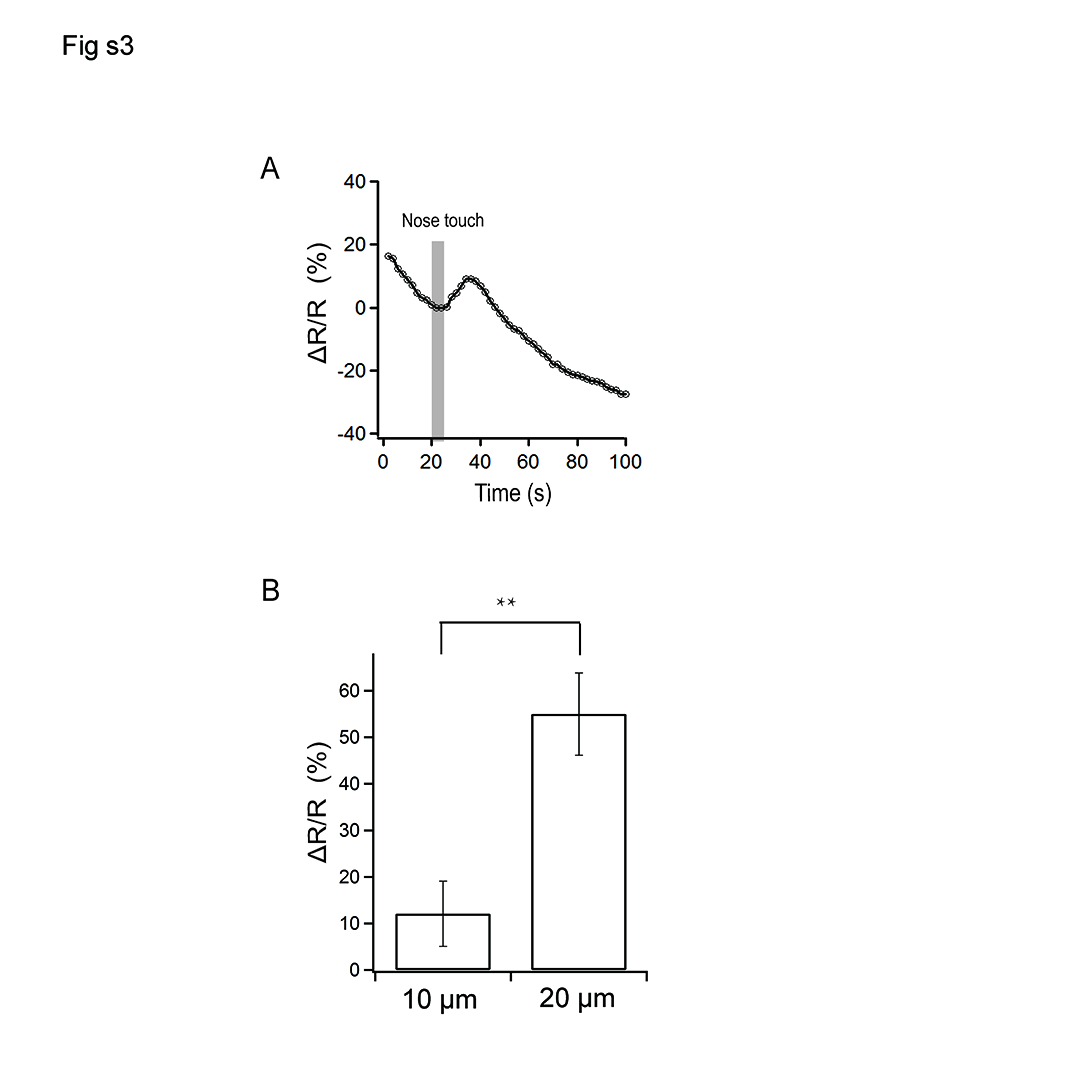

Supplement: S3 Fig — (A)Representative GCaMP/mCherry ratio changes induced by 10 μm displacement (2 Hz, 5 s). (B)Statistical summary of GCaMP/mCherry ratio changes induced by 10 μm and 15 μm displacement (2 Hz, 5 s) (mean± s.e.m.; n≥4). (TIF) [file pone.0117114.s004.tif]
